# Tuberculosis After Gastrectomy, Plasmatic Concentration of Antitubercular Drugs

**DOI:** 10.4084/MJHID.2012.007

**Published:** 2012-01-24

**Authors:** Giuseppe Vittorio De Socio, Antonio D’Avolio, Alessio Sgrelli, Lorena Baietto, Lisa Malincarne, Giovanni Di Perri, Franco Baldelli

**Affiliations:** 1 Department of Infectious Diseases “Santa Maria della Misericordia” Hospital, University of Perugia, Perugia Italy; 2 Laboratory of Clinical Pharmacology and Pharmacogenetic, Department of Infectious Diseases, University of Turin, Amedeo di Savoia Hospital, Turin, Italy

## Abstract

We report pharmacokinetic data on two gastrectomized, patients affected by tuberculosis. Drugs plasmatic concentrations were measured after seven days of oral therapy by a validated high performance liquid chromatography-mass spectrometry (HPLC-MS) method and the area under the concentration-time-curve (AUC) over 24 hours (AUC_0–24_) was calculated. A sub-therapeutic level of isoniazid was found in a patient with total gastrectomy with a C_max_ of 0,395 mg\L and AUC_0–24_ level of 4.75 hr*mg/L. The level of the other antitubercular drugs was adequate. These findings support the need to monitor anti tubercular drug levels to facilitate early detection of therapeutic failure, above all in patients treated with isoniazid and with potential problems on oral drugs absorption.

## Introduction

Little is known of antitubercular treatment in gastrectomized patients. By a MEDLINE search we found no evidence in the literature regarding the antitubercular drugs absorption after gastrectomy. Total and partial gastric resection alters the anatomy and secretory activity of the gastrointestinal tract. It might be expected that the consequences of such changes should affect the pharmacokinetics, especially concerning the absorption of orally administered drugs that are primarily absorbed in the stomach or duodenum leading to sub-therapeutic drugs level.

## Case Studies and Methods

We report here the case of two consecutive gastrectomized patients affected by tuberculosis. To estimate the degree to which absorption is impaired, the drug levels were measured in duplicate plasma samples. The patients were fasting at the time of drugs intake. Plasma sample levels were measured by a validated high performance liquid chromatography-mass spectrometry (HPLC-MS) method with the following lower limit of quantification (LOQ) and detection (LOD): 0.015 mg/L (LOQ) and 0.007 mg/L (LOD) for isoniazid; 0.195 mg/L (LOQ) and 0.048 mg/L (LOD) for pyrazinamide; 0.019 mg/L (LOQ) and 0.008 mg/L (LOD) for rifampin; 0.020 mg/L (LOQ) and 0.010 mg/L (LOD) for ethambutol. Non-compartmental pharmacokinetic analysis of the data was performed using Kinetica version 5.0 software (Thermo Fisher Scientific). The area under the concentration-time curve (AUC) over 24 hours (AUC_0–24_) in plasma was calculated by the linear-log trapezoidal rule, using the same C_trough_ concentration for time 0 and 24 and applying a limited sampling model.^1^

### Case 1

A 68-year-old, HIV negative was affected by tubercular (TB) epididymitis. His previous medical history was remarkable for a total gastrectomy with Roux-en-Y gastric bypass procedure performed five years before, due to gastric cancer. His renal and liver function was normal. His weight was 65Kg. He was treated with standard oral anti TB therapy including isoniazid 300 mg daily, rifampin 600 mg daily, ethambutol 1200 mg daily and pyrazinamide 2000 mg daily, vitamin B6 300 mg daily. His chronic medications were: enalapril 20 mg daily, amlodipine 5 mg daily, ticlopidine 500 mg daily. Therefore plasma concentrations of anti TB drugs were measured after seven days of drug intake in the hospital. Plasma samples were collected before the drug intake to measure C_trough_ and after 2 hours to measure C_max_ (peak plasma concentration). We found very low C_max_ and AUC_0–24_ levels of isoniazid (0,395 mg\L and 4.75 hr*mg/L respectively), not attributable to patient’s anti-hypertensive and anti-tubercular drug-drug interaction. The results of plasma concentrations, AUC_0–24_ and the therapeutic expected range are shown in the following **table**. The patient obtained slow clinical improvement.

### Case 2

A 65-year-old HIV negative woman was affected by pulmonary tuberculosis. She underwent a partial gastrectomy for a gastric ulcer 15 years earlier. Her weight was 39 Kg; she was treated with standard anti TB therapy including isoniazid 250 mg daily, rifampin 600 mg daily, ethambutol 1000 mg daily and pyrazinamide 1000 mg daily, vitamin B6 300 mg daily. The drugs were administered intra venous (iv) in the first 15 days and oral subsequently (pyrazinamide and vitamin B6 were always administered orally). No other drugs were taken by this patient. We studied the plasmatic drugs concentration in the course of iv and oral therapy. Plasma samples were collected before the daily drug intake to measure C_trough_ and after 2 hours and 5 hours to measure C_max_. The results of plasma concentrations, AUC_0–24_ during oral therapy are shown in the table. During iv therapy the measured AUC_0–24_ was very similar from the one reported in the course of oral treatment: isoniazid 26.70 hr*mg/L, rifampin 154.87 hr*mg/L, ethambutol 32.48 hr*mg/L. The patient obtained adequate clinical response.

## Discussion

To our knowledge, data regarding the first line antitubercular drugs absorption after gastrectomy are scantily. Overall the measured plasmatic antitubercular drugs in gastrectomized patients were generally adequate. We observed that a patient undergoing a complete gastric resection followed by a Roux-en-Y gastric bypass procedure (case 1) had poor isoniazid plasmatic level. In the second case with the patient who had undergone a partial gastric resection, the absorption of isoniazid was right and comparable to iv administration. Rifampin, ethambutol and pyrazinamide in both patients always had suitable level.

Obviously, a single case study has several limitations principally linked to individual variability, thus we can’t draw conclusions. In addition, in case 1 we have calculated the AUC_0–24_ from two determinations only according to a validated limited sampling model,^1^ and assuming C_max_ appropriate after two hours of drugs intake. Even considering these limitations, we believe the observation is important at least for two reasons: firstly, it is known that gastrectomy patients have greater risk of having tuberculosis in later life and preventive therapy is mainly based on isoniazid administration;^2^,^3^ secondly, low serum concentration of antitubercular drugs have been associated with treatment failure, relapse and drug resistance.^4^

Although the clinical significance of low concentration should be defined, it may be necessary to optimise drug dosages by TDM, especially in patients with an inadequate clinical response.

These findings support the need to monitor anti tubercular drug levels to facilitate early detection of therapeutic failure above all in patients with potential problems on oral drugs absorption.

## Figures and Tables

**Table 1:** Anti-tubercular drugs plasma concentration and therapeutic range references.

Anti tubercular drugs	Time after dose, h	Patient 1	Patient 1	Patient 1 AUC_0–24_ (hr*mg/L)	Patient 2	Patient 2	Patient 2 AUC_0–24_ (hr*mg/L)	Expected therapeutic references mg/L	Expected range of AUC_0–24_ (hr*mg/L)
	(C_max_, C_trough_)	dose mg/day	Drug plasma concentration mg/L		dose mg/day	Drug plasma concentration mg/L		(C_max_ and Cut-off C_max_)	
Isoniazid	2	300 (4.6)*	0.395	4.75	250 (6.4)*	5.05	37.61	C_max_ 9–15[5] Cut-off Cmax 3.0 [5]	10.52 – 111.80 [8,9]
	5		ND			2.2			
	24		< 0.007 (LOD)			0.03			
Rifampin	2	600 (9.2)*	9.671	116.40	600 (15)*	12.67	145.37	C_max_ 9.65–24.99 [6] Cut-off Cmax 8.0 [7]	47.40.–142.73 [6]
	5		ND			9.64			
	24		0.029			0.72			
ethambutol	2	1200 (18.5)*	4.547	54.58	1000 (25)*	7.08	57.43	Cut-off C_max_ 2.0 [6]	ND
	5		ND			2.94			
	24		< 0.010 (LOD)			0.70			
pyrazinamide	2	2000 (30.7)*	47.269	5832.50	1000 (25)*	32.53	337.22	C_max_ 21.70–42.64 [6] Cut-off Cmax 35.0 [7]	303.35–541.36 [6]
	5		ND			21.54			
	24		13.346			1.84			

Area under the concentration-time curve (AUC_0–24_); lower limit of detection (LOD);

* mg per Kg per day; ND=unavailable.
